# Whole body insulin sensitivity is increased in systemic sclerosis

**DOI:** 10.1371/journal.pone.0283283

**Published:** 2023-03-30

**Authors:** Jacopo Ciaffi, Piero Ruscitti, Ilenia Di Cola, Viktoriya Pavlych, Noemi Italiano, Martina Gentile, Tom Huizinga, Jeska K. de Vries-Bouwstra, Francesco Ursini, Paola Cipriani

**Affiliations:** 1 Medicine & Rheumatology Unit, IRCCS Istituto Ortopedico Rizzoli (IOR), Bologna, Italy; 2 Department of Biotechnological and Applied Clinical Sciences, University of L’Aquila, L’Aquila, Italy; 3 Rheumatology Department, Leiden University Medical Center, Leiden, The Netherlands; 4 Department of Biomedical and Neuromotor Sciences (DIBINEM), Alma Mater Studiorum University of Bologna, Bologna, Italy; Bolu Abant İzzet Baysal University: Bolu Abant Izzet Baysal Universitesi, TURKEY

## Abstract

**Objectives:**

In the present study, we aimed to evaluate whole-body insulin sensitivity in systemic sclerosis (SSc) patients and to compare the results with controls with no autoimmune rheumatic disease (non-ARD) and with patients affected by rheumatoid arthritis (RA).

**Methods:**

In all patients and controls, oral glucose tolerance test (OGTT) was performed according to the World Health Organization (WHO) recommendations. Plasma glucose and insulin concentrations were measured at time 0 and then after 30, 60, 90, and 120 minutes. Whole-body insulin sensitivity (ISI), insulinogenic index (IGI), oral disposition index (ODI), and insulin resistance (HOMA-IR) were estimated accordingly.

**Results:**

A total of 41 SSc patients were evaluated and, for comparison, 41 individuals with RA and 82 non-ARD control patients were recruited. OGTT yielded a proportion of normotolerant individuals among SSc patients higher than in RA controls (p = 0.040) but lower than in the non-ARD group (p = 0.028). The ISI was significantly higher in SSc patients compared with RA controls (p <0.001) and with non-ARD patients (p <0.001). Significant differences emerged also when analysing the HOMA-IR, which was lower in SSc patients than in RA (p <0.001) and non-ARD (p <0.001) groups. Additionally, IGI was lower in SSc patients compared with RA (p = 0.011) and with non-ARD controls (p <0.001), whereas ODI was not significantly different between groups.

**Conclusions:**

Interestingly, we found that SSc patients are more insulin sensitive than those with RA and even than individuals without inflammatory diseases. In contrast, no significant difference was found in terms of β-cell function.

## Introduction

Systemic sclerosis (SSc) is an autoimmune connective tissue disease characterized by microvascular dysfunction and excessive collagen deposition leading to fibrosis of skin and internal organs [1]. SSc is considered a rare disease [2] and its pathogenesis is poorly understood, with a possible role of genetic, hormonal, environmental and occupational factors [1, 3, 4]. The extent of skin involvement defines two major clinical subtypes, namely limited cutaneous (lcSSc) and diffuse cutaneous SSc (dcSSc) [5]. However, the clinical presentation of SSc is highly heterogeneous, ranging from mild forms to severe, life-threatening disease associated with significant morbidity and reduced quality of life [6, 7]. Mortality rate is high in SSc; available literature shows a pooled standardized mortality ratio of 3.45 [8], mainly contributed by interstitial lung disease and pulmonary arterial hypertension [9].

Patients with autoimmune rheumatic diseases (ARD) have an increased risk of cardiovascular events [10] resulting from the pernicious interplay between systemic inflammation and increased prevalence of traditional cardiovascular disease (CVD) risk factors, as clearly demonstrated in rheumatoid arthritis (RA) [11] and systemic lupus erythematosus (SLE) [12]. Similarly, emerging data, although less robust, suggest an increased risk of cardiovascular events in SSc [13].

Type 2 diabetes (T2D) is a strong, independent CVD risk factor and its role has been extensively demonstrated in the general population [14]. The risk of T2D is increased in RA [15] and other inflammatory diseases such as psoriasis [16], mainly as a consequence of inflammation-induced and drug-induced insulin resistance [17].

Insulin resistance and ß-cell dysfunction are the main mechanisms in the pathophysiology of T2D [18]. Insulin is secreted by pancreatic β-cells in response to rising arterial concentrations of glucose. Under conditions of insulin resistance, β-cells are stimulated to secrete more insulin than under conditions of normal insulin sensitivity. With time, insufficient insulin secretion, glucolipotoxicity, and obesity-related inflammation, result in hyperglycaemia and, finally, T2D.

Interestingly, in a large Australian cohort, Ngian et al. [19] found a lower prevalence of T2D in SSc patients than in controls drawn from a national, longitudinal, population-based study. Consistently, in a retrospective cohort study, Tseng et al. [20] demonstrated a reduced incidence of T2D in SSc in both sexes compared to controls. These results were further corroborated by Ursini et al. [21] who confirmed a significantly lower prevalence of T2D in SSc than in the general Italian population.

However, to the best of our knowledge, the potential mechanisms underlying the negative association between SSc and T2D have not been investigated yet.

Whole-body sensitivity to insulin is directly measured through the euglycemic hyperinsulinemic clamp technique, but this quantification method is extremely difficult to apply in daily clinical practice [22]. As a valid alternative, the oral glucose tolerance test (OGTT) is commonly used to assess glucose homeostasis *in vivo*. Different indices have been proposed to obtain information about insulin sensitivity from OGTT results, but adequate validation against euglycemic insulin clamp has seldom been performed [23]. Currently, a reliable estimate of whole-body insulin sensitivity is provided by the index proposed by Matsuda, [23] easily calculated from measurements of dynamic plasma glucose and insulin concentrations obtained during the OGTT.

Therefore, in the present study, we aimed to evaluate whole-body insulin sensitivity in SSc patients and to compare the results with controls with no autoimmune rheumatic disease (non-ARD) and with patients affected by RA.

## Materials and methods

### Study population

The study population was composed of consecutive adult patients fulfilling the 2013 ACR/EULAR classification criteria for SSc [24] followed at the Rheumatology Division of the University of L’Aquila from January 2022 to June 2022. For comparison, control patients without autoimmune diseases (osteoarthritis or fibromyalgia), and patients with RA, attending the involved centres in the same time and satisfying the 2010 ACR/EULAR classification criteria [25], were included in the study.

### Screening for T2D and inclusion/exclusion criteria

All individuals were screened for T2D before inclusion. Screening was performed according to the American Diabetes Association (ADA) 2018 recommendations [26]. Briefly, blood samples were collected after overnight fasting. Fasting plasma glucose (FPG) and glycated haemoglobin (HbA1c) were checked. If FPG ≥ 126mg/dL or HbA1c ≥ 6.5% (48 mmol/mol), patients were classified as having T2D and excluded from the study. Patients with abnormal FPG results (≥ 100mg/dL) and HbA1c <6.5% were re-evaluated one week later and, in case of inconsistent results, a third evaluation was performed after an additional week. Patients were excluded if any of the values obtained during the three-step evaluation exceeded the threshold for T2D (FPG ≥ 126mg/dL or HbA1c ≥ 6.5% or 48 mmol/mol).

Individuals with a past diagnosis of diabetes, polycystic ovary syndrome, neoplastic diseases, and severe gastrointestinal involvement, defined according to previously described criteria [27], were excluded from the study. Additional exclusion criteria were current use of glucocorticoids and past or current treatment with insulin-sensitizing agents (e.g., metformin, thiazolidinediones).

The research was conducted in compliance with the Declaration of Helsinki and its latest amendments. The study was approved by the local Ethics Committee (*Comitato Etico Azienda Sanitaria Locale 1Avezzano-Sulmona-L’Aquila*, *L’Aquila*, Italy; protocol number 015408/17). Written informed consent was obtained from all patients.

### Demographic and clinical data

Demographic and clinical characteristics were gathered at time of inclusion in the study. In patients with SSc or RA, disease duration was defined as time since the diagnosis. For patients with SSc, disease subset, presence of interstitial lung disease (ILD) or pulmonary arterial hypertension (PAH), history of digital ulcers and modified Rodnan skin score (mRSS) were also recorded.

To assess whether insulin sensitivity was correlated with the presence and the severity of gastrointestinal symptoms, the Italian version of the University of California, Los Angeles Scleroderma Clinical Trial Consortium Gastrointestinal Tract Instrument 2.0 (UCLA-GIT) was administered to all SSc patients [28]. This SSc-specific instrument was developed to assess the severity of gastrointestinal symptoms and their impact on quality of life and consists of 34 items grouped in seven domains: reflux, distention/bloating, diarrhoea, faecal soilage, constipation, emotional wellbeing and social functioning [29]. All domains range from 0 to 3, except diarrhoea and constipation, which are scored respectively from 0 to 2 and from 0 to 2.5. Total score is the average of six domains (excluding constipation) and ranges from 0 to 2.83. Higher values indicate a greater impact of gastrointestinal involvement on quality of life.

### Evaluation of whole-body insulin sensitivity

In all patients, OGTT was performed according to the World Health Organization (WHO) recommendations [30]. After overnight fast, the patient was invited to drink a solution with 75g of anhydrous glucose dissolved in 200mL of water over a time of 5 minutes. Blood samples were collected at time 0 and then after 30, 60, 90, and 120minutes. Plasma glucose and insulin concentrations were measured at each time point.

Glucose tolerance was classified according to the American Diabetes Association [26]. Patients were considered normotolerant if their fasting plasma glucose (FPG) was <100 mg/dL and their 2h post-load glucose was <140 mg/dL. Impaired fasting glucose (IFG) was defined if FPG between 100 and 125 mg/dL and a 2h post-load glucose <140 mg/dL. Patients with FPG <100 mg/dl and 2h post-load glucose 140–199mg/dL were classified as having impaired glucose tolerance (IGT), while those with 2h post-load glucose ≥200 mg/dL were diagnosed with T2D.

Whole-body insulin sensitivity (ISI) was estimated from glucose and insulin concentration obtained during OGTT using the equation proposed by Matsuda, which is validated against euglycemic insulin clamp and provides reliable estimates of glucose homeostasis [23]:

$$ISI\;=\frac{10.000}{\sqrt{G0\times I0\times G\;mean\times I\;mean}}$$

where *G0* is FPG in mg/dL, *I0* is fasting plasma insulin mcU/L, *G mean* is the mean plasma glucose during OGTT in mg/dL and *I mean* is mean plasma insulin during OGTT in mcU/L.

Insulinogenic index (IGI), a measure of early phase insulin secretion, defined as the ratio of the increment of insulin to that of plasma glucose 30 minutes after a glucose load, was calculated with the formula:

$$IGI=\frac{\Delta I0-30\;min}{\Delta G0-30\;min}$$

Oral disposition index (ODI), a measure of β-cell function integrated with insulin sensitivity, was calculated with the formula: ODI = IGI × ISI

ODI=IGI*ISI

In addition, the original homeostasis model assessment of insulin resistance (HOMA-IR) by Matthews et al. [31] was calculated from overnight fasting insulin and glucose values, with the following formula:

$$HOMA-IR=\frac{G0\;*\;I0}{405}$$

### Statistical analysis

Data are expressed as mean ± standard deviation (SD), median [25^th^-75^th^ percentile] or number (percentage) as appropriate. Independent samples T-test test and Mann-Whitney U test were used to compare means of continuous variables between two groups, respectively for normally and non-normally distributed continuous variable, while chi-squared test was used to compare categorical variables. The Spearman’s correlation coefficient (rho) was used to analyse the univariate association between insulin sensitivity and demographic or clinical characteristics. Significant variables, i.e., those with a p-value <0.05 in correlation analysis, were included in a multiple linear regression model to evaluate the independent association between ISI and mRSS, expressed as R^2^ coefficient and p-value. All statistical analyses were performed using the Statistical Package for Social Sciences (SPSS) software ver. 26.0 (IBM, Armonk, NY, USA).

## Results

### General characteristics of the study population

A total of 64 SSc patients were consecutively seen during the study period, of which 41 satisfied the inclusion criteria. Reasons for exclusion were: refuse to participate in the study (n = 9), past diagnosis of T2D (n = 1), neoplastic diseases (n = 2), polycystic ovary syndrome (n = 2), severe gastrointestinal involvement (n = 3), current use of glucocorticoids (n = 6). For comparison, 41 individuals with RA not taking glucocorticoids (22/41 treated with methotrexate alone, 1/41 treated with sulfasalazine, 1/41 treated with leflunomide, 5/41 treated with TNF inhibitors combination therapy, 5/41 treated with abatacept combination therapy, 7/41 treatment naive) and 82 non-ARD control patients, satisfying the same inclusion/exclusion criteria, were recruited.

General characteristics of the study population are summarized in Table 1. Of the 41 patients with SSc, 34 (82.9%) were female. SSc patients were significantly older (59.4 ± 13.1 vs 53.8 ± 11.4 years, p = 0.041) and had lower body mass index (BMI) (24.0 ± 3.4 vs 27.8 ± 6.5 kg/m^2^, p = 0.002) than controls with RA, but had comparable characteristics with non-ARD patients. Regarding the disease subset, 65.9% of patients had lcSSc, 22% had dcSSc and 12.2% had the sine scleroderma subset. Mean mRSS was 8.7 ± 5.9 and mean UCLA-GIT score was 0.59 ± 0.44.

**Table 1:** Characteristics of the study population.

	SSc (n = 41) ^a^	RA (n = 41) ^b^	Non-ARD ^c^ (n = 82)	p-value
**Female, n (%)**	34 (82.9)	27 (65.9)	65 (79.3)	0.077 ^ab^ 0.629 ^ac^
**Age, years**	59.4 ± 13.1	53.8 ± 11.4	58.3 ± 8.4	0.041 ^ab^ 0.546 ^ac^
**BMI, kg/m^2^**	24.0 ± 3.4	27.8 ± 6.5	24.3 ± 2.2	0.002 ^ab^ 0.559 ^ac^
**Systemic hypertension, n (%)**	10 ± 24.4	16 ± 39	21 ± 25.6	0.154 ^ab^ 0.883 ^ac^
**Ever smokers, n (%)**	16 (39)	15 (36.6)	24 (29.3)	0.820 ^ab^ 0.276 ^ac^
**Disease duration, years**	7.4 ± 3.6	8.4 ± 4.1		
***SSc characteristics***				
**Diffuse cutaneous SSc, n (%)**	9 (22)	-	-	-
**Limited cutaneous SSc, n (%)**	27 (65.9)	-	-	-
**Sine scleroderma SSc, n (%)**	5 (12.2)	-	-	-
**ILD, n (%)**	13 (31.7)	-	-	-
**PAH, n (%)**	2 (4.9)	-	-	-
**Digital ulcers, n (%)**	8 (19.5)	-	-	-
**mRSS**	8.7 ± 5.9	-	-	-
**UCLA GIT score**	0.59 ± 0.44	-	-	-

### OGTT and insulin *sensitivity* in patients with systemic sclerosis and controls

The results of OGTT are reported in Table 2. Mean FPG was 84.0 ± 9.8 mg/dL in SSc patients, 87.7 ± 10.6 mg/dL in RA (SSc vs RA p = 0.102) and 88.8 ± 7.2 mg/dL in the non-ARD group (SSc vs non-ARD p = 0.008). Mean fasting plasma insulin was 6.9 ± 6.7 mcU/mL in SSc, 12.1 ± 7.9 mcU/mL in RA (SSc vs RA p = 0.002) and 9.0 ± 5.9 mcU/mL in non-ARD (SSc vs non-ARD p = 0.083) patients.

**Table 2:** Glucose metabolism in SSc and controls.

	SSc (n = 41) ^a^	RA (n = 41) ^b^	Non-ARD ^c^ (n = 82)	p-value
**Fasting plasma glucose, mg/dl**	84.0 (9.8)	87.7 (10.6)	88.8 (7.2)	0.102 ^ab^ 0.008 ^ac^
**Fasting plasma insulin, mcU/ml**	6.9 (6.7)	12.1 (7.9)	9.0 (5.9)	0.002 ^ab^ 0.083 ^ac^
**Normal glucose tolerance, n (%)**	35 (85.4)	27 (65.9)	79 (96.3)	0.040 ^ab^ 0.028 ^ac^
**Impaired fasting glucose, n (%)**	1 (2.4)	2 (4.9)	1 (1.2)	0.556 ^ab^ 0.614 ^ac^
**Impaired glucose tolerance, n (%)**	4 (9.8)	8 (19.5)	2 (2.4)	0.211 ^ab^ 0.076 ^ac^
**Impaired fasting glucose and impaired glucose tolerance, n (%)**	1 (2.4)	1 (2.4)	0	1.000 ^ab^ 0.156 ^ac^
**Diabetes, n (%)**	0	3 (7.3)	0	0.078 ^ab^ 1.000 ^ac^
**HOMA-IR**	0.98 (0.66–1.52)	2.02 (1.36–3.63)	1.74 (1.28–2.28)	< 0.001 ^ab^ < 0.001 ^ac^
**ISI**	8.01 (4.85–12.62)	3.39 (1.93–6.14)	4.38 (3.39–6.37)	< 0.001 ^ab^ < 0.001 ^ac^
**IGI**	0.59 (0.43–0.96)	1.01 (0.52–1.58)	1.00 (0.62–1.60)	0.011 ^ab^ < 0.001 ^ac^
**ODI**	3.73 (1.86–12.83)	2.85 (1.84–5.68)	4.50 (2.94–6.96)	0.146 ^ab^ 0.584 ^ac^

OGTT yielded a proportion of normotolerant individuals among SSc patients higher than in RA controls (85.4% vs 65.9%, p = 0.040) but lower than in the non-ARD group (85.4% vs 96.3%, p = 0.028). No difference in prevalence of IFG, IGT, IFG+IGT or T2D was observed between SSc and RA or non-ARD controls. However, based on the OGTT results, three patients with RA received a new diagnosis of T2D.

The ISI was significantly higher in SSc patients compared with RA controls [8.01 (4.85–12.62) vs 3.39 (1.93–6.14), p <0.001] and with non-ARD patients [8.01 (4.85–12.62) vs 4.38 (3.39–6.37), p <0.001]. In further subanalysis, a significantly higher value of ISI was demonstrated when dcSSc where compared with RA patients [11.60 (8.59–20.13) vs 3.39 (1.93–6.14), p < 0.001]. The difference was still evident when ln-transformed ISI was used as a dependent variable in a multivariate analysis of covariance (ANCOVA) model corrected for age and BMI (SSc vs RA p < 0.001; SSc vs non-ARD p < 0.001).

Stratifying RA patients according to disease activity, the difference in ISI was still maintained [SSc vs RA moderate/severe disease activity (n = 26): 8.01 (4.85–12.62) vs 2.93 (1.73–4.52), p <0.001; SSc vs RA low disease activity/remission (n = 15): 8.01 (4.85–12.62) vs 4.90 (2.02–7.63); p = 0.019].

Significant differences emerged also analysing the HOMA-IR, which was lower in SSc patients than in RA [0.98 (0.66–1.52) vs 2.02 (1.36–3.63), p <0.001] and non-ARD [0.98 (0.66–1.52) vs 1.74 (1.28–2.28), p <0.001] groups. Additionally, IGI was lower in SSc patients compared with RA [0.59 (0.43–0.96) vs 1.01 (0.52–1.58), p = 0.011] and with non-ARD controls [0.59 (0.43–0.96) vs 1.00 (0.62–1.60), p <0.001], while ODI was not significantly different between groups.

**Fig 1:**
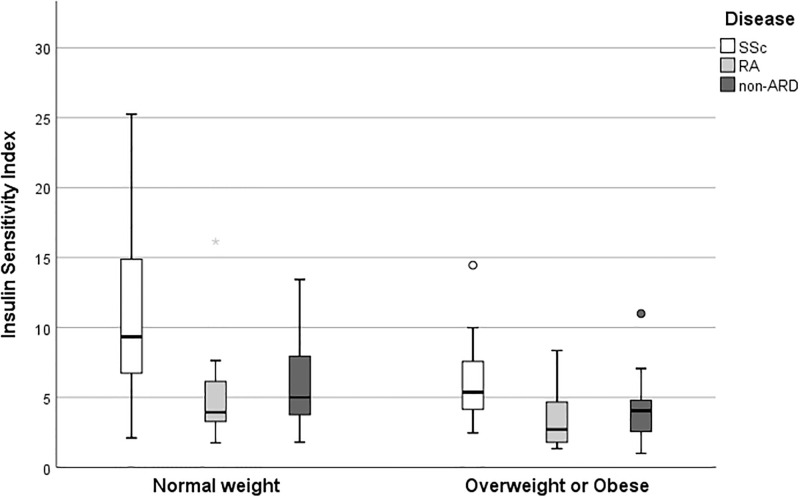
Box-plot distribution of insulin sensitivity index in normal weight (BMI 18.5–24.9) and overweight or obese (BMI ≥ 35) patients. Boxes represent the range from the 25^th^ to the 75^th^ percentile of each group’s distribution of values, with horizontal black lines denoting median values. Vertical lines correspond to adjacent values, which are the most extreme values within 1.5 interquartile range of the 25^th^ and 75^th^ percentile of each group. Circles represent outliers, while asterisks are extreme outliers.

Considering the potentially relevant role of weight, ISI differences between groups were also evaluated in the population stratified according to BMI ranges (Fig 1). In normal weight individuals (BMI from 18.5 to 24.9), median ISI was higher in the SSc group than in RA (p = 0.007) and non-ARD controls (p <0.001). Also in overweight and obese cases (BMI ≥25) ISI was higher in the SSc group than in RA (p = 0.003) and non-ARD controls (p = 0.017). Underweight patients (n = 2 in SSc, n = 1 in RA, n = 0 in non-ARD) were excluded from this analysis. No subanalysis on the subgroup of obese patients was performed since only three patients in the SSc group had BMI ≥30 kg/m^2^; however, the comparison of overweight patients between groups did not change significantly the overall results [SSc 5.45 (3.03–8.62) kg/m^2^ Vs RA 3.60 (1.77–7.09) kg/m^2^, p = 0.083; SSc 5.45 (3.03–8.62) kg/m^2^ Vs non-ARD 4.05 (2.53–4.89) kg/m^2^, p 0.055]. Similar results when ISI was compared amongst patients classified as NGT on the basis of OGTT results [SSc 8.53 (4.93–14.45) vs RA 3.78 (2.31–7.03), p < 0.0001; SSc 8.53 (4.93–14.45) vs non-ARD 4.53 (3.46–6.65), p < 0.0001].

In the SSc group, patients with dcSSc had significantly higher ISI than individuals with lcSSc [11.60 (8.59–20.13) vs 6.58 (4.91–11.63), p = 0.027] or sine scleroderma [11.60 (8.59–20.13) vs 3.93 (2.94–9.28), p = 0.028], while no difference was observed between lcSSc and sine scleroderma [6.58 (4.91–11.63) vs 3.93 (2.94–9.28), p = 0.243].

In univariate analysis, ISI was significantly correlated with BMI (rho = -0.468, p = 0.002), fasting insulin (rho = -0.715, p <0.001), and mRSS (rho = 0.422, p = 0.006), while no association emerged with age (rho = -0.213, p = 0.181), FPG (rho = -0.248, p = 0.118), disease duration (rho = -0.208, p = 0.191), and UCLA-GIT score (rho = -0.101, p = 0.529) in SSc patients. In multiple linear regression model adjusted for BMI and fasting plasma insulin, the association between ISI and mRSS was lost (r^2^ = 0.27, p = 0.095).

## Discussion

Several studies demonstrated that ARDs are frequently associated with insulin resistance [17, 32], an early step in the natural history of T2D [18, 19]. Despite sharing the inflammatory background, accumulating clinical data suggest that the prevalence of T2D is low in SSc patients [19–21]. However, the mechanisms underlying this phenomenon have not been fully explored.

On this background, we aimed at evaluating glucose metabolism in SSc patients. Interestingly, we found that SSc patients are more insulin sensitive than those with RA and even than individuals without inflammatory diseases. In contrast, no significant difference was found in terms of β-cell function, as expressed by ODI. Consistently, differences in ISI were maintained in different subanalysis carried on to extrapolate the confounding effect of BMI, SSc subset, and glucose tolerance status. Notably, higher ISI in SSc vs RA patients was evident despite in the latter population a proportion of patients was treated with molecules known to improve insulin sensitivity such as TNF inhibitors or abatacept [33–35].

The strength of our study relies on the use of ISI proposed by Matsuda [23], a measure that provides a reliable estimate of whole-body insulin sensitivity obtained from dynamic values of glucose and insulin, in contrast to other widely used indices (e.g., HOMA-IR) derived from fasting values that are believed to primarily reflect hepatic insulin sensitivity [36]. Consequently, this instrument allows to catch a potential contribution from metabolically active tissues other than liver, including adipose tissue, skeletal muscles, and possibly skin in this specific setting.

One intuitive explanation for the enhanced insulin sensitivity observed in SSc patients is the potential role of gastrointestinal involvement. Gastrointestinal symptoms are common in SSc, involving approximately 90% of patients [37], with a prevalence of malnutrition over 10% [38]. On the other hand, there is a well-recognized relationship between obesity and insulin resistance [39], with insulin sensitivity improving when obese patients lose weight and return to normal weight [40]. Therefore, in SSc patients, gastrointestinal involvement may promote weight loss that, in turn, favours an improvement in obesity-dependent insulin resistance. Further, gastrointestinal dysmotility in SSc is commonly treated with a combination of prokinetic agents and dietary modifications [37], including eating smaller meals more frequently. A similar eating pattern has been associated with a lower risk of developing T2D and with improvement of glycaemic control in patients with T2D and prediabetes [41].

In attempt to account for this confounder, patients with severe gastrointestinal involvement were *a priori* excluded from the study and analyses were adjusted for BMI and for the degree of gastrointestinal involvement assessed through the UCLA-GIT score. Although the contribution of gastrointestinal involvement and associated eating pattern/behaviour cannot be extrapolated from our data, other fascinating disease-specific mechanisms can be postulated to affect insulin sensitivity in SSc patients.

From an immunometabolic perspective, serum levels of interleukin-13 (IL-13), interleukin-10 (IL-10), and tumor necrosis factor-related apoptosis-inducing ligand (TRAIL) are increased in SSc patients and these molecules have been proposed to exert a protective effect against T2D [42–45]. Further, fibroblasts from SSc show a unique metabolic derangement, including a glucose-demanding increase in glycolysis [46]. Moreover, tissue hypoxia stimulates the expression of glucose transporter GLUT-1 [47], which is increased in the skin of SSc patients [44], thus potentially promoting glucose uptake by skin. This mechanism may dictate a shift from prevalently hepatic to extra-hepatic (i.e., cutaneous) glucose clearance. Indeed, increased in vivo skin and soft tissues glucose avidity has been described in isolated reports by using positron emission tomography (PET) [48] and emerging, although limited, evidence suggests that soft tissue uptake correlate with mRSS and can surrogate the extent of skin involvement [49]. In this context, it is possible also to speculate that redirecting glucose to the liver by repurposing well known therapeutics may promote a clinical benefit mediated by a “relative glucose starvation” of the skin. In support of this hypothesis, oral metformin has been demonstrated to improve skin thickness and fibroblasts accumulation in the bleomycin-induced mouse model of skin sclerosis [50, 51].

Accordingly, in our cohort, we observed higher insulin sensitivity in patients with more extensive skin involvement (dcSSc vs lcSSc); mRSS was positively correlated with ISI although the association was lost in a multivariate model including BMI and fasting insulin. Therefore, the role of skin in using glucose may be complex and influenced by the relative amount of other insulin-sensitive tissues (such as adipose tissue) or by baseline production of insulin, yet the role of the small sample size cannot be excluded.

Further, liver involvement is not infrequent in SSc [52]. In patients with no insulin resistance, liver fibrosis can impair gluconeogenesis resulting in lower fasting plasma glucose levels [53]. Similarly, in SSc patients, subclinical liver involvement may determine lower baseline glucose levels resulting in a higher ISI. Although this mechanism is expected to affect mainly other surrogate measures such as HOMA-IR relying solely on fasting values, we should acknowledge that fasting glucose is still present in the ISI formula.

In conclusion, to the best of our knowledge, this is the first time that whole-body insulin sensitivity is assessed in SSc by using a validated and reliable method, thus providing relevant and unique data to the current limited knowledge about glucose homeostasis in SSc. However, further research, including euglycemic insulin clamp and PET studies, are warranted to entirely unveil the mechanisms underlying the higher insulin sensitivity and the lower prevalence of T2D in SSc patients.
